# Nurses’ attitude towards oral care and their practicing level for hospitalized patients in Orotta National Referral Hospital, Asmara-Eritrea: a cross-sectional study

**DOI:** 10.1186/s12912-020-00457-3

**Published:** 2020-07-10

**Authors:** Zewdi Amanuel Dagnew, Isayas Afewerki Abraham, Ghirmay Ghebreigziabher Beraki, Eyasu Habte Tesfamariam, Sibyl Mittler, Yobiel Zemhret Tesfamichael

**Affiliations:** 1 Emergency and Critical Care Unit, Department of Nursing, Orotta College of Medicine and Health Sciences, Asmara, Eritrea; 2 Midwifery Unit, Department of Nursing, Orotta College of Medicine and Health Sciences, Asmara, Eritrea; 3 Epidemiology and Biostatistics Unit, Department of Statistics, College of Sciences, Eritrean Institute of Technology, MaiNefhi, Eritrea; 4 Anesthesia Unit, Department of Nursing, Orotta College of Medicine and Health Sciences, Asmara, Eritrea; 5 Department of Dialysis, Orotta National Referral Hospital, Asmara, Eritrea

**Keywords:** Nurses, Oral care, Attitude, Practice, Hospitalized patients, Orotta hospital, Eritrea

## Abstract

**Background:**

Effective and routine mouth care is necessary for hospitalized patients as it helps to maintain the health of oral cavity and overall health. However, oral care is often overlooked and not prioritized in daily activity plan of nurses even when oral problems are apparent. Therefore, the aim of this study was to assess nurses’ attitude towards oral care and their practicing level for hospitalized patients.

**Methods:**

A cross-sectional study design was conducted in adult medical-surgical department of Orotta hospital from December 2017 to January 2018. Data was collected from all (*N* = 73) diploma and associate nurses through face to face interview using a pretested and structured questionnaire. Kruskal-Wallis, Mann-Whitney U tests and spearman rank correlation coefficient tools were performed to analyze the data using SPSS (Version 22).

**Results:**

Out of the 79 participants, 73 completed the interview successfully with a response rate of 92.4%. Of the total, 56.2% were diploma nurses and 43.9% were associate nurses. The median attitude score was 68.89/100 (IQR = 48.89). The majority (94.5%) of the nurses agreed that oral cavity assessment is nurse’s responsibility and 94.5% reported adequate training is needed to provide quality oral care. On the other hand, the median practice score was 50.00/100 (IQR = 17.86). Majority of the participants (76.7%) did not perform routine oral health assessment. Almost all (98.4%) used gauze and normal saline for oral care. Practice score was significantly different across the various wards (*p* < 0.001), however, it was not significantly correlated with attitude (*p* = 0.646).

**Conclusions:**

The participants had poor level of oral care practice to hospitalized patients, nevertheless, they had favourable attitude. Therefore, Orotta National Referral Hospital needs to give further effort to train the nursing staff, ensure the availability of adequate oral care equipment and provide clear guidelines regarding oral care of hospitalized patients.

## Background

Oral care is one of the fundamental nursing procedures that have adverse effect on patient well-being and general health. Patients’ oral hygiene is defined as the practice of keeping the oral cavity healthy through cleaning of gums, teeth, tongue, lips and dentures [1]. Effective and routine oral care is necessary for maintaining the health of oral cavity and overall health of hospitalized patients [2]. On the other hand, poor oral hygiene causes oral discomfort, pain, and effect on chewing and swallowing that affect nutritional intake. It affects the quality of life and has a negative effect on nutritional status, increased vulnerability to gum and respiratory infections [3].

In most healthy adults’ oral cavity consist about 350 harmless aerobic microorganisms [4]. However, in hospitalized patients, these nonpathogenic microbes are potential risk to ship to pathogenic if oral care is not given within 48 h of admission. These virulent organisms form dental plaque in the oral cavity which becomes a source of bacteria and toxins [4–7]. If the plaque content is dislodged and get an access to enter the blood stream or the lungs it may cause cardiac or lung infections. Moreover, lately, poor oral hygiene has been shown to be a possible independent risk factor for hypertension [5].

In hospitalized patients’ Hospital Associated Pneumonia (HAP) is the first leading cause of death and the third most common cause of infection [4]. In industrialized countries, hospital-acquired infections like HAP and Ventilator Associated Pneumonia (VAP) are the main causes of morbidity and mortality with a death rate of around 21.8, 62 and 62.07% in USA, Canada and India, respectively [8–10]. In a condition where a patient is under endotracheal tube (ETT)/ventilator, the ETT acts as a direct conduit for the dental plaque to enter into the lungs, which might cause VAP [4, 5, 11]. Ventilated patients have higher risk (9 to 27%) to develop pneumonia with a mortality rate of 36 to 60% of all hospital related deaths [4]. A study done in Florida revealed that oral hygiene was significantly associated with prevention of HAP in patients without endotracheal tube [5]. Therefore meticulous oral care is recommended for hospitalized patients to enhance comfort and reduce the incidence of nosocomial pneumonia [12].

In critical care units or in medically compromised patients, the mouth needs special attention [2, 13, 14]. In these patients oral cavity should be assessed, cleansed with a soft bristled tooth brush and documented properly at least in every shift [4]. However, oral care is often overlooked and not prioritized in daily activity plan of nurses even when oral problems are apparent [12, 15]. Even if oral care is performed it’s done in a substandard way just by swabbing the mouth with gauze and normal saline solely for comfort measure [13, 14, 16, 17]. Moreover, many studies showed that oral care is often seen as a difficult and unpleasant task by nurses which compromises its practice [14, 16, 18].

The role of nurses in maintaining the oral health and wellbeing of hospitalized patients is undeniable [16]. Having this in mind it is essential that nurses should be aware of evidence-based information and use of standard protocols to deliver appropriate oral care in their setting. The first step is to change the attitude of the nurses from viewing oral care solely as a comfort measure; to oral care as an obligation to help to improve nursing practice, create positive social change by improving the quality of care provided to patients, and improve patient outcomes by providing comfort and decreasing the risk of aspiration [19]. This could be achieved by enhancing their practice via improving the nursing curricula, providing on the job training and workshops, and equipping the health care setup.

Nurses, as well-established, are the principal health-care providers for patients admitted to hospitals and provision of oral care is one of their duties [20]. In African studies, existing data shows that majority of nurses had favorable attitude towards prioritizing oral care in their nursing care plan. Hospital-based data are available for specific sites in Sudan (97.4%), Nigeria (94.3%), Egypt (81%) and South Africa (97.9%) [3, 16, 17, 21]. In contrast, unfavorable attitude towards oral care has been reported in some studies – 84% in Menoufia University study [22]. On the other hand, in studies done in Egypt (78%) and South Africa (57.6%) majority of the nurses used gauze swabs with mouth wash to clean the mouth [16, 17]. Moreover, in the study done in Menoufia University, 100% of the nurses had poor practice of oral care [22].

In Eritrea, oral care is not considered as an essential care for patients in the hospital. Subsequently, it was not done routinely. Even if it was performed, the practice was unsatisfactory (it was given once a day using gauze and normal saline or clean water). And generally, to date, there is no documented evidence that shows the attitude and practice of nurses or related studies towards patient oral care and its associated factors in Eritrea. This is therefore the first study to investigate the attitude and practice regarding oral care delivery for hospitalized patients among nurses working in Orotta hospital.

Therefore, the aim of this study was to assess the nurses’ attitude towards oral care and their practicing level for hospitalized patients in adult medical–surgical department of Orotta hospital and identify any associated factors. The finding of the study might help in improving quality of nursing care, particularly oral care and practice, among nurses. This may enhance oral health in Eritrea where there are few dentists. In addition, the findings may provide essential data for subsequent studies.

## Methods

### Study design and setting

A cross-sectional design was used to conduct the study. The study was carried out in Orotta National Referral Hospital (ONRH), the only national referral hospital in Eritrea. ONRH is situated in Asmara, the capital city of Eritrea. The population of Eritrea (as of 2017) was estimated at about 3,700,167. The country has three-tier system that includes primary, secondary, and tertiary health services. ONRH is one of the tertiary level hospitals which has begun its service on 17th June, 2004. It was designed for medical-surgical clinical services with 517 beds and had established diagnostic facilities. As it is a national referral hospital, it provides services to residents of the capital city and all those patients referred from other regional referral hospitals. The focus of the study area was the adult medical-surgical inpatient department which includes; medical, surgical, intensive care unit (ICU), ear nose and throat (ENT), emergency and recovery wards with a total of 88 nursing staff.

### Study recruitment

The study was conducted from December 2017 to January 2018. No sample size determination method was applied, because complete enumeration of the nurses working in the study area was performed. There were a total of 79 diploma and associate nurses who provide direct bedside nursing care in the ONRH. However, six participants did not meet the inclusion criteria (four were non-respondents and two were on annual leave), hence 73 nurses were enrolled in the study.

### Data collection tools and techniques

The questionnaire and scoring system were adopted and modified by the researchers to suit the hospital setting after reviewing similar studies [3, 22]. The questionnaire was divided into three parts. The first part (7-questions) was designed to record the general demographic data of participants. The second part comprised nine questions to assess participants’ attitudes toward oral care of hospitalized patients. In the last part of the questionnaire, participants were asked 20 questions related to their current practice regarding oral care of patients at their current work place in the hospital. Then, data was collected through face to face interview with a structured questionnaire by the researchers.

### Variable measurement

#### Attitude towards oral care

In order to measure the attitude of the nurses towards oral care, nine questions were used. Each item was responded using five-point Likert type (5 = strongly agree, 4 = somewhat agree, 3 = neutral, 2 = somewhat disagree, 1 = strongly disagree). The composite attitude scores were totaled from nine questions (six positively worded and three negatively worded) giving an ideal minimum and maximum scores of 9 and 45 respectively. The responses for the negatively worded questions were reversed while computing the composite attitude scores. As it might be difficult to interpret and understand the raw composite scores, they were converted to percentage. With increase in score, the level of favorable attitude increases.

#### Practice on oral care

A composite practice scores were obtained by totaling 19 items. Each correct answer of the item was assigned 1 mark and 0 otherwise, leading to minimum and maximum possible scores of 6 and 28 respectively. The computed composite score was further transformed to percentage. With increase in score of practice, the level of good practice also increases.

### Data collection procedure

Data was collected after ethical approval had been obtained by the research and ethics committee of Asmara College of Health Sciences (ACHS) and Eritrean Ministry of Health. The researchers met the medical director and matron of the ONRH to explain the purpose of the study, the clinical implication, and the data collection process. After selecting the data collectors, a mini work shop was held to explain the purpose of the research, data collection process and question by question guidance for the questionnaire. Later on, the data collectors were assigned to specific study sites of the ONRH to proceed with the interview after getting the consent from the participants. The average time taken for completing the interview was around 20–30 min. Finally, the researchers revised each questionnaire for its completion.

### Validity and reliability

The English version questionnaire was reviewed by a panel of experts in nursing and dentistry at Asmara College of Health Sciences and ONRH. Through this process, the face and content validity of the questionnaire was guaranteed. The reliability of the attitude scale was satisfactory enough in other previous studies (Cronbach’s Alpha = 0.84) [22].

### Pre-test

The questionnaire was pre-tested among ten nurses in similar hospital named; Hazhaz Regional Referral Hospital, to evaluate the clarity, ease in understanding, applicability, and to estimate the time needed to complete the interview 1 month before the study period. The interview was done face-to-face by four researchers. The pre-designed questions that were not easily understood by the interviewee were simplified after pre-testing the questionnaire.

### Data quality control measures

The data collectors were told to promptly check any questionnaire after they fill it out, on spot. Then, the collected data were once again cleaned by the researchers for its completeness and any inconsistencies. Furthermore, in order to minimize the data entry errors, an entry program developed with Census and Survey Processing System (CSPro, Version 7.0) software package, that automatically checks the data structure of the file using skip patterns and range of valid values, was used.

### Data analysis

Data analysis was done using SPSS-version 22. Frequency (percentage), mean (SD), and median (IQR) were used to describe the variables, as appropriate. Normality of the data was evaluated before the start of the analysis using Fisher’s measures of skewness and kurtosis. Difference in attitude and practice scores across characteristics of the participants for non-normally distributed data were analyzed using Kruskal-Wallis and Mann-Whitney U tests. The correlation between attitude and practice was evaluated using spearman rank correlation coefficient. Post-hoc analysis was also done for significant associations found in Mann-Whitney U test. Additionally, descriptive statistics such as frequency distribution was used to provide an overall and comprehensible presentation of the data. A *p*-value of less than or equal to 0.05 was considered statistically significant throughout the analyses.

### Operational definition

#### Diploma nurses

Diploma nurses are those who are graduated at diploma level after completion of three theoretical and practical years.

#### Associate nurses

Associate nurses are those who are graduated at certificate level after completion of one and half theoretical and practical years.

#### Oral care

Is the use of a protocol that comprises, an initial assessment, tooth brushing, use of alcohol free antiseptic, avoiding the use of lemon, elevation of the head, suctioning and daily assessment.

## Results

### Characteristics of the participants

A total of 79 participants were eligible, out of these, 73 participants (92.4% response rate) were enrolled in the study. More than half (56.2%) of the participants were diploma nurses and the rest (43.85%) were associate nurses. The median age and work experience of the participants were (Md = 26, IQR = 5, Min. =21, Max. =54) and (Md = 4, IQR = 5, Min. =1, Max. =19) respectively. The participants were working in Medical (27.4%), Surgical (19.2%), Emergency (19.2%), ICU (17.8%), Recovery (11%) and ENT (5.5%) wards.

### Attitude on oral care

Almost all (94.5%) nurses agreed that assessment of oral cavity was nurse’s responsibility. Moreover, 89% of the participants agreed that oral care is high priority of nursing activity, however, 78.1 and 54.8% disagreed that cleaning the mouth is unpleasant and difficult activity respectively. The majority (94.5%) of the participants were positive about the need for sufficient training and oral care guideline (97.3%). Besides, 71.2% disagreed with provision of enough supplies and equipment for quality oral care (Table 1).

**Table 1:** Percentage distribution of the nurses’ attitude towards oral care (*n* = 73)

Attitude	Strongly/somewhat agree n (%)	Neutral n (%)	Strongly/somewhat disagree n (%)
It is nurses responsibility to assess the oral status	69 (94.5)	1 (1.4)	3 (4.1)
Oral care is high priority	65 (89.0)	6 (8.2)	2 (2.7)
Cleaning the oral cavity is an unpleasant task^a^	13 (17.8)	3 (4.1)	57 (78.1)
The mouth of patients gets worse no matter I do^a^	2 (2.7)	4 (5.5)	67 (91.8)
I need enough training to provide oral care	69 (94.5)	4 (5.5)	0 (0)
The oral cavity is difficult to clean^a^	29 (39.7)	4 (5.5)	40 (54.8)
Ventilated/comatose patients should be given special attention	73 (100)	0 (0)	0 (0)
I need an oral care guideline to provide quality oral care	71 (97.3)	1 (1.4)	1 (1.4)
I have enough supplies and equipment to provide oral care	17 (23.3)	4 (5.5)	52 (71.2)

^a^ Negatively worded questions

The median attitude score was 68.89/100 (IQR = 8.89, Minimum = 44.44, and Maximum = 80.00), showing a favorable attitude towards the oral care of the patients by the nurses. Kruskal-Wallies and Mann-Whitney U tests revealed that the categories of participants’ characteristics did not have significant (*p* ≥ 0.05) difference in attitude scores (Table 2).

**Table 2:** Difference in scores of attitude towards oral care across different categories of participants’ characteristics (*N* = 73)

Demographics	Md (IQR)	Test Statistic (λ^**2**^, df / M-W, Z/ρ)	***P***-value
Age group			
20 to 25	68.89 (6.67)	λ^2^ = 0.453, df = 2	0.797
26 to 30	68.89 (11.11)		
> 30	70.00 (14.44)		
Sex			
Male	68.89 (7.22)	M-W = 394, Z = -1.30	0.192
Female	68.89 (8.89)		
Level of nursing education			
Associate Nurse	68.89 (10.56)	M-W = 578.5, Z = 0.869	0.385
Diploma nurse	68.89 (7.78)		
Experience			
1 to 2	68.89 (8.89)	λ^2^ = 0.215, df = 2	0.898
3 to 7	68.89 (8.89)		
> 7	68.89 (11.67)		
Ward currently working			
Medical	68.89 (6.67)	λ^2^ = 3.07, df = 4	0.546
Surgical	67.78 (11.11)		
Emergency	68.89 (6.11)		
ICU	68.89 (11.11)		
Recovery	72.22 (10.00)		

### Practice of oral care

More than three quarter (76.7%) of the participants did not perform routine oral health assessment, however, 82.4% performed assessment to unconscious patients and 58.8% to ventilated patients. Regarding the availability of guideline/procedure for oral care, all (100%) responded negatively. The majority of participants (83.6%) give oral care to patients, of these only 26.2% give oral care routinely. Of the total, 57.4% provided oral care for non-intubated patients once a day whereas 8.2% provided oral care to intubated patients greater than three times a day (every 4 h). Besides, participants who do oral care; 100% cleaned the tooth, 85.2% cleaned the gums and 88.5% cleaned tongue. Almost all (98.4%) of those who practice oral care used gauze and normal saline while only 9.8% used patients own adult tooth brush to clean the oral cavity. Majority (79.3%) raised the bed to a correct degree (30–45 degree) when performing oral care. Of which, 62.3% used moisturizer (Vaseline) after doing oral care. However, all (100%) participants had never used chlorohexidine (CHX) or other type of mouth wash for oral care (Table 3).

**Table 3:** Percentage distribution of the participants’ practice of oral care (*N* = 73)

Practice	Frequency	Percent
Routine assessment of oral health		
Yes	17	23.3
No	56	76.7
To whom do you perform oral assessment		
To all patients	3	17.6
To ventilated patients	10	58.8
To unconscious patients only	14	82.4
Conduct an initial admission assessment		
Yes	5	6.8
No	12	16.4
Discuss oral health status and management for oral care		
Always	8	11.3
Sometimes	6	8.2
Never	3	4.1
Availability of guideline		
Yes	0	0
No	73	100
Give oral care		
Yes	61	83.6
No	12	16.4
Frequency of oral care to patients		
Routinely	16	26.2
Sometimes	21	34.4
Rarely	24	39.3
Performance for non-intubated patients		
Once a day	35	57.4
Twice a day	18	29.5
Three times a day	4	6.6
Greater than 3 times a day	4	6.6
Performance for intubated patients		
Once a day	2	3.3
Twice a day	4	6.6
Three times a day	3	4.96
Greater than 3 times a day	5	8.2
Not relevant	47	77
Oral cavity cleaned		
Tooth	61	100
Gums	52	85.2
Tongue	54	88.5
Performance of oral care to hospitalized patients		
Suctioning only	15	24.6
Gauze and normal saline	60	98.4
Adult tooth brush	6	9.8
Raise head while giving oral care		
Yes	58	95.1
No	3	4.9
To what degree?		
15	1	1.7
30 to 45	46	79.3
60	1	1.7
90	10	13.7
Ever used Chlorohexidine mouth wash		
Yes	0	0
No	61	100
Do you apply moisturizer (Vaseline) on the lips?		
Yes	38	62.3
No	23	37.7

The median oral care practice score was 50.00/100 (IQR = 17.86, Minimum = 0 and Maximum = 96.43). Practice of oral care scores were not significantly different in the various age groups (*p* = 0.094), sex (*p* = 0.479), level of nursing education (*p* = 0.535), and years of experience (*p* = 0.152). Moreover, practice was not substantially correlated with attitude (*p* = 0.646). However, significantly different practice score was observed across the various wards (*p* < 0.001) (Table 4).

**Table 4:** Difference in scores of oral care practice across different categories of participants’ characteristics (*N* = 73)

Demographics	Md (IQR)	Test Statistic (λ^**2**^, df / M-W, Z/ρ)	***P***-value
Age group			
20 to 25	46.43 (18.75)	λ^2^ = 4.72, df = 2	0.094
26 to 30	50.00 (21.43)		
> 30	41.07 (58.04)		
Sex			
Male	53.57 (16.96)	M-W = 440, Z = -708	0.479
Female	50.00 (17.86)		
Level of Education			
Health assistant	50.00 (23.21)	M-W = 600.5, Z = -0.620	0.535
Diploma nurse	50.00 (19.64)		
Experience			
1 to 2	46.43(41.07)	λ^2^ = 3.77, df = 2	0.152
3 to 7	53.57 (21.43)		
> 7	50.00 (22.32)		
Ward currently working			
Medical	44.64 (39.29)	λ^2^ = 38.18, df = 4	< 0.001
Surgical	50.00 (7.14)		
Emergency	42.86 (26.79)		
ICU	75.00 (14.29)		
Recovery	62.50 (22.32)		
Attitude Score		Spearman ρ = 0.055	0.646

Post-hoc analysis in the difference of oral care practice across various wards was performed using Mann-Whitney U test. Except five pairs, namely, medical & emergency, medical & ENT, surgical & emergency, emergency & ENT, and ICU & recovery, all other pairs of wards had significantly different oral care practice (Table 5).

**Table 5:** Post-Hoc analysis in differences of the oral care practice across different wards

Ward	Medical	Surgical	Emergency	ICU	Recovery	ENT
Medical	–	0.010	0.616	< 0.001	< 0.001	0.056
Surgical	–	–	0.329	< 0.001	0.020	0.005
Emergency	–	–	–	< 0.001	0.005	0.079
ICU	–	–	–	–	0.064	0.001
Recovery	–	–	–	–	–	0.004

## Discussion

Oral care of hospitalized patients is an important preventive measure that aims to maintain and promote the health of oral and dental tissues [20]. In this context, majority of the nurses agreed that assessment of oral cavity as part of their responsibility. The role of the nurse as a care provider of oral care for hospitalized patients cannot be underestimated. Such practice should become a routine part of their daily nursing duties. However, nurses will not be able to deliver quality oral care unless they are well-trained and educated in this respect. This should be coupled with building a favorable attitude among nurses toward the importance of oral cavity assessment for the welfare of hospitalized patients [20]. This finding is also supported by a study done in India and Ethiopia [14, 23].

Moreover, more than three quarter of the participants agreed that oral care is a high priority area in nursing care. This finding is in agreement to the studies done in Saudi Arabia, Pakistan, South Africa and Egypt which revealed a similar attitude among a majority of nurses [16–18, 20]. However, it was in disagreement with a finding from a study done in Taiwan, which demonstrated that nurses attach low priority to oral care when compared to other nursing activities for hospitalized patients, while tracing the reasons for this attitude of nursing to insufficient time to provide oral care due to their huge workload [24].

In the present study more than three quarter of the nurses disagreed that cleaning the oral cavity is unpleasant task. This finding is supported by the studies done in Pakistan, India and South Africa in which more than half of the participants agreed or strongly agreed that it’s a pleasant task [14, 16, 18].

Training and education related to oral care of hospitalized patients should be an essential part of nursing academic programs and continuous educational courses [20]. In the present study, almost all of the nurses agreed that they need continuous training and education to provide effective oral care. This finding is consistent with a study done in Menoufia university hospital, Egypt [22], but inconsistent with a study done in India [14]. Furthermore, hospitals should issue clear guidelines, policies and protocols for the assessment and management of oral health for hospitalized patients, although its presence does not guarantee compliance [18, 20]. In our study a large number of the nurses agreed that presence of oral care protocol in their respective ward is basic in providing oral care up-to standard. This finding is consistent to the study done in Pakistan and South Africa [16, 18]. Moreover, in our study, majority of the participants agreed that they do not have enough equipment to provide oral care, in line to a studies done in Egypt and Sudan [3, 22]. However, a study done in South Africa reported the converse [16].

The findings of this study reveal a very favorable attitude among the vast majority of the surveyed nurses toward the provision of oral care for hospitalized patients. This finding is similar to a study report in Ethiopia [23]. These findings may reflect the huge efforts that have been paid by nursing staff and may increase the likelihood of accomplishing the procedure, however, favorable attitude alone is not enough. Therefore, nursing care must be reinforced with compulsory learning and oral care rehearsals.

Looking to the practice of nurses, the majority of nurses did not perform routine oral cavity assessment, and all nurses reported that they did not have guideline for oral care procedure. These finding are inconsistent with the Pakistan, Nigeria and Ethiopia studies [18, 21, 23]. Proper assessment and early intervention often prevent serious complications before they can compromise therapeutic outcomes. Nurses are able to recognize early changes in patient status and problems [21]. However, the findings of this investigation indicate a problem on the level of current practice regarding oral care of hospitalized patients in Orotta hospital.

The American Association of Critical Care nurses guidelines recommend routine brushing at least twice a day using a soft pediatric or adult toothbrush [25]. Primary prevention through tooth brushing has paramount importance in the prevention of dental health problems [26]. In the present study, more than half of the nurses give oral care to patients, but only few did it routinely. This is inconsistent to the study done in Sudan in which majority of the nurses performed oral care twice a day [3]. Moreover, the majority of study participants performed oral care only once a day using gauze and normal saline which is similar with the studies done in Croatia, Egypt and Iran [17, 25, 27]. However, this is inconsistent with the study done in Brazil, in which more than half of the participants used tooth brush to provide oral care [2]. Chlorhexidine (CHX) is a very effective wide spectrum antiseptic agent with rare occurrence of side effects [25]. However, most of the study participants have neither used CHX nor heard the name CHX. Moreover, the use of toothbrush was limited. This outcome indicates that oral care was largely inadequate in this setting. This may be due to lack of adequate knowledge, equipment and supplies as well as shortage of time.

The poor practice of oral care in Orotta hospital can be attributed to the absence of guideline and lack of effective oral care equipment and supplies. The use of oral assessment tools and evidence-based oral care practice guidelines have been shown to result in significantly improved patient oral assessment scores [11]. In this study, the majority of nurses considered the correct positioning of patient during oral care. Application of lip moisturizers (brought by family members) after oral care was also reported. This finding is comparable to the studies done in Pakistan and Egypt [18, 22]. The aforesaid shortcomings need to be addressed in a further research to provide solutions and recommendations for improvements of oral care delivery for hospitalized patients in Orotta hospital.

The attitude level of the nurses was not significantly associated with the participants’ characteristics, whereas, the score of practice was significantly different across the various wards. This finding is similar to a study report in Ethiopia [23]. This result appears to suggest that majority of the nurses working in ICU and Emergency were highly involved with critically ill patients and patients who need immediate intervention and treatments. These may lead them to read and update themselves. Notably, the practice scores were not correlated with the attitude. Thus, even though they have favorable attitude, lack of knowledge, shortage of time, and insufficient equipment may contribute to inadequate practice.

The current study was undertaken only in Orotta National Referral Hospital, so the results cannot be generalized to cover all diploma and associate nurses in Eritrea, and this can be considered as a limitation. Thus, it can be argued that the results of this study provide a rather important insight for health services planners about the views and practices of nursing staff regarding the delivery of oral care for hospitalized patients in Orotta hospital, Eritrea. To obtain a clearer picture, future national survey is highly recommended.

## Conclusions

The oral health of hospitalized patients is affected by several factors like medical illness, medications, supporting devices (ETT and nasogastric tube) and immunity. Assessing the attitude and practice of oral care is primarily the responsibility of nurses. The result of this study shows that nurses have a favorable attitude toward delivery of oral care. However, the oral care practice in Orotta National Referral Hospital was poor. Therefore, it needs for additional training on oral care. Availability of adequate oral care equipment and provision of clear guidelines on oral care of hospitalized patients should be prioritized.

### Recommendations

Based on the findings of this study, the following recommendations were made:

1. As the study shows poor practices to oral care, it is recommended that there must be teaching sessions for the nurses in the forms of seminars, workshops and symposiums to enhance the attitude and practice. This will improve knowledge and enhance positive attitude towards oral care practice.
2. Orotta hospital should endeavor to provide adequate equipment’s and supplies for the delivery of oral care in the hospital.
3. At organizational level there must be a guideline, protocol and policy to asses and provide oral care to the newly admitted patients and retained patients as early as possible.

## Supplementary information

**Additional file 1: Part 1.** Questionnaire on background information. **Part 2.** Questions on attitude. **Part 3.** Questions on oral care practice.

## Data Availability

The complete data set supporting the conclusions of this article is available from the corresponding author and can be accessed upon a reasonable request.

## Abbreviations

CDC: Centers for Disease Control
CHX: Chlorhexidine
ENT: Ear, Nose and Throat
ETT: Endotracheal tube
HAP: Hospital Associated Pneumonia
ICU: Intensive Care Unit
IQR: Interquartile Range
ONRH: Orotta National Referral Hospital
SPSS: Statistical Package for Social Science
VAP: Ventilator Associated Pneumonia
WHO: World Health Organization
