# Supplementary material for: Nurses’ attitude towards oral care and their practicing level for hospitalized patients in Orotta National Referral Hospital, Asmara-Eritrea: a cross-sectional study
Source: BMC Nurs. 2020 Jul 10;19:63. doi: 10.1186/s12912-020-00457-3 (PMC7348104; doi:10.1186/s12912-020-00457-3)
Supplement: Supplementary file 1 — Additional file 1: Part 1. Questionnaire on background information. Part 2. Questions on attitude. Part 3. Questions on oral care practice. [file 12912_2020_457_MOESM1_ESM.docx]

**Additional file 1**

**Part 1:** Questionnaire on background information

| **NO** | **Question** | **Coding** | **Skip** |
| --- | --- | --- | --- |
| 101 | Age | ________ |  |
| 102 | Sex | 1. Male 2. Female |  |
| 103 | What is your highest level of education? | 1. Health assistant 2. Diploma nurse |  |
| 104 | The ward within which you are working | 1. Medical Ward 2. Surgical Ward 3. Emergency Ward 4. ICU ward 5. Recovery Ward |  |
| 105 | What is your work experience in Orotta national referral hospital? | 1. <1year 2. 1-5year 3. 6-10year 4. >10year |  |

**Part 2:** Questions on attitude

| **NO** | **Question** | **Coding** | **Skip** |
| --- | --- | --- | --- |
| 201 | It is nurses’ responsibility to assess the oral status of the patients. | 1. strongly agree 2. somewhat agree 3. neutral 4. somewhat disagree 5. strongly disagree |  |
| 202 | Oral care is high priority. | 1. strongly agree 2. somewhat agree 3. neutral 4. somewhat disagree 5. strongly disagree |  |
| 203 | Cleaning the oral cavity is an unpleasant task. | 1. strongly agree 2. somewhat agree 3. neutral 4. somewhat disagree 5. strongly disagree |  |
| 204 | The mouth of critically ill patients gets worse no matter what I do. | 1. strongly agree 2. somewhat agree 3. neutral 4. somewhat disagree 5. strongly disagree |  |
| 205 | I need additional training to provide oral care. | 1. strongly agree 2. somewhat agree 3. neutral 4. somewhat disagree 5. strongly disagree |  |
| 206 | The oral cavity is difficult to clean. | 1. strongly agree 2. somewhat agree 3. neutral 4. somewhat disagree 5. strongly disagree |  |
| 207 | Ventilated or comatose patients should be given special attention in doing oral care. | 1. strongly agree 2. somewhat agree 3. neutral 4. somewhat disagree 5. strongly disagree |  |
| 208 | I need an oral care guideline to provide quality oral care. | 1. strongly agree 2. somewhat agree 3. neutral 4. Somewhat disagree 5. Strongly disagree |  |
| 209 | I have enough supplies and equipment’s to provide oral care. | 1. strongly agree 2. somewhat agree 3. neutral 4. somewhat disagree 5. strongly disagree |  |

**Part 3: questions on oral care practice**

| **NO** | **Question** | **Coding** | **Skip** |
| --- | --- | --- | --- |
| 301 | Do you have a tool to assess the oral cavity of a patient? | 1. Yes 2. No 3. I don’t know |  |
| 302 | Do you assess the oral health of a patient routinely? | 1. Yes 2. No 3. I don’t know | **→** 407 |
| 303 | If your answer to question number “401’’ is “YES”, on what proportion of your patients do you carry out oral cavity assessment? | 1. To all patients 2. To ventilated patients 3. To unconscious patients only |  |
| 304 | Do you assess for dental plaque, dry mouth, bleeding gums, reddened gums, mouth ulcer during your assessment? | 1. Yes 2. No |  |
| 305 | Do you conduct an initial admission assessment on patient’s oral health? | 1. Yes 2. No |  |
| 306 | Do you discuss the oral health status and management for oral care deficits during the nurses’ rounds? | 1. Always 2. Sometimes 3. Never |  |
| 307 | Do you give oral care to your patients? | 1. Yes 2. No | **→** 420 |
| 308 | On what basis do you give oral care to your patients | 1. Routinely 2. Sometimes 3. Rarely |  |
| 309 | Do you have oral care guideline or protocol in your ward? | 1. Yes 2. No |  |

| 310 | How often per day do you perform oral care for non-intubated patient? | 1. Once a day 2. Twice a day 3. Three times a day 4. greater than 3/day |  |
| --- | --- | --- | --- |
| 311 | How often per day do you perform oral care for intubated patient? | 1. Once a day 2. Twice a day 3. Three times a day 4. greater than 3/day | **Not relevant** |
| 312 | Which part of the oral cavity do you clean? | 1. Tooth 2. Gums 3. Tongue 4. All |  |
| 313 | How long does it take you to perform oral care for a patient? | 1. 1min 2. 2 - 4min 3. 5-10 min 4. >10min |  |
| 314 | How do you perform the oral care to hospitalized patients? | 1. Adult tooth brush 2. Pediatric tooth brush 3. Electric Tooth Brush 4. Gauze with normal saline 5. Suctioning only |  |
| 315 | Do you raise the head of the bed while giving oral care? | 1. Yes 2. No | **→** 417 |
| 316 | If your answer to question number **415** is **Yes** to what degree? | 1. 15 degrees 2. 30-45 degrees 3. 60 degrees 4. 90 degrees |  |
| 317 | Do you use suction machine when providing oral care, if the patient is unable to spit out secretions? | 1. Yes 2. No |  |
| 318 | Have you ever used Chlorohexidine mouth wash for oral care? | 1. Yes 2. No 3. Don’t know 4. If other, please specify________ |  |
| 319 | Do you apply moisturizer (like Vaseline) on the lips after oral care? | 1. Yes 2. No | **Finnish** |
